# A Presentiment

**Published:** 1890-07

**Authors:** 


### A PRESENTIMENT.

Recently, a young lady at the Palmer House, Chicago, fearing that there was something wrong with her father, who was in another room, got up in the night only to hear him fall dead. The facts as stated in the daily papers, are as follows:

"Papa! papa! let me in!" was the cry which awakened a number of the guests and startled the night watchman upon one of the floors at the Palmer House, at 3 o'clock this morning. They were uttered by Miss Perkins, a twenty-year old daughter of H. O. Perkins, of 107 Arlington place, Cleveland, a varnish manufacturer of the firm of Blakeslee & Co. There was no response to the girl's agonized cry. The watchman soon had the door open, however, and there upon the floor laid the girl's father, dead.

The grief of the daughter was extremely pitiful. Her father was a large, exceptionally handsome man of 45 years. He had been traveling in the West for several months, and was on his way home. His daughter had also been away from home since the holidays, attending school. Her father sent for her to meet him here so that they could go home together and give the wife and mother a pleasant surprise. They arrived on Saturday morning, and were assigned adjoining rooms, at the Palmer House. They intended leaving for home Saturday evening, but Mr. Perkins concluded to remain over till this morning, in order to show his daughter a little more of the city. Yesterday, they called upon several friends, among them being T. O. Bolger, with the real estate firm of B. F. Jacobs & Co. Mr. Perkins complained of feeling unwell, and attributed it to the fact that he had stopped smoking a few nights ago.

This morning his daughter awakened with a consciousness that something ailed her father. She went to the door communicating with the two rooms to listen. Just as she reached it she heard a sound as of a falling body striking the floor, and then all was still. This was what caused her startling cry. The worst fears of the poor girl were realized. The body was removed to Klaner's undertaking establishment, and this afternoon will be taken by the daughter and some friends to Cleveland. Neighbors of Mr. Perkins in Cleveland were telegraphed to break the news to Mrs. Perkins.—*Relig. Philo. Journal.*

